# Mediastinal lesions across the age spectrum: a clinicopathological comparison between pediatric and adult patients

**DOI:** 10.18632/oncotarget.17201

**Published:** 2017-04-18

**Authors:** Tingting Liu, Lika'a Fasih Y. Al-Kzayer, Xiao Xie, Hua Fan, Shamil Naji Sarsam, Yozo Nakazawa, Lei Chen

**Affiliations:** ^1^ Department of Pediatric Hematology/Oncology, Xinhua Hospital, Shanghai Jiaotong University School of Medicine, Shanghai, China; ^2^ Department of Pediatrics, Shinshu University School of Medicine, Matsumoto, Japan; ^3^ Department of Thoracic Cardiovascular Surgery, Xinhua Hospital, Shanghai Jiaotong University School of Medicine, Shanghai, China; ^4^ Department of Radiology, Xinhua Hospital, Shanghai Jiaotong University School of Medicine, Shanghai, China; ^5^ Department of Radiology, Ibn Al-Nafees Hospital, Manama, Kingdom of Bahrain; ^6^ Department of Pathology, Xinhua Hospital, Shanghai Jiaotong University School of Medicine, Shanghai, China

**Keywords:** mediastinal lesions, age distribution, histopathological distribution, the mediastinum

## Abstract

The objective of this study was to identify the differences in histopathological distribution and clinical features of mediastinal lesions (MLs) across the age spectrum in Chinese series of patients and to compare with the available literature. A total of 409 cases of MLs, including 137 pediatric and 272 adult patients from a single institution, was reviewed and categorized into groups according to age. Among the 409 cases, the age showed a bimodal distribution with an increased incidence of MLs among (< 10 year) and (60–< 70 year) age groups. Thymic lesions, neurogenic tumors, and cysts made up 57% of MLs among the 409 cases. A significantly higher frequency was found for neurogenic tumors, germ cell tumors, mesenchymal tumors, and lymphatic lesions, (*p* < 0.01) for all, in pediatric population compared to adults. On the contrary, frequencies of thymic lesions and metastatic carcinomas were significantly higher in adults compared to pediatric category, (*p* < 0.01) for both. Overall, 41.6% were asymptomatic, however, pediatric patients showed a significantly higher incidence of cough and fever, (*p* < 0.01) for both, and dyspnea (*p* = 0.02), than adults. Whereas adult subset showed a significantly higher incidence of chest pain (*p* = 0.02), or oppression (*p* < 0.01), than pediatric counterpart. In conclusion, the age spectrum was the factor that influenced the histopathological distribution and the clinical presentation of MLs in Chinese series of patients. Such differences might be considered in the differential diagnosis and therapeutic approach for adult as well as pediatric patients with MLs. Furthermore, our study was comparable to the literature in terms of MLs frequencies.

## INTRODUCTION

Mediastinal lesions (MLs) can develop from structures that are normally located in the mediastinum or that pass through the mediastinum during development, as well as from metastases of malignancies that arise elsewhere in the body. MLs span a wide histopathological and radiological spectrum, including any mass, benign or malignant, infectious or reactive, and they could occur throughout lifespan. The most frequent lesions encountered in the mediastinum are thymomas, neurogenic tumors (NTs) and benign cysts, altogether representing 60% of patients with MLs. Age-related differences of tumor biology, host characteristics, or treatment protocol exist in MLs. Histopathological distribution, location, symptomatology and prevalence of malignancy are different between adult and pediatric populations [1–6]. Because of the heterogeneity of MLs, descriptive data on age patterns and incidence trends of particular histopathological type are limited.

Age distribution and clinical features, together with their anatomic location from the mediastinum provide important diagnostic information for MLs. Davis et al, had demonstrated in a series of 400 consecutive patients with primary MLs that 50% of symptomatic patients had a malignant neoplasm before 1967 compared with 62% after that year [7]. Of note, a large percentage of MLs produce no symptoms and are found incidentally during chest radiograph or imaging studies of the thorax performed for other reasons [8]. The distinct clinical characteristics in MLs between children and adults are well reported, however, it is less clear whether the age spectrum has effects on the clinicopathological features.

Herein, we present the differences in histopathological distribution of MLs across the age spectrum in Chinese patients with comparison to the available literature, and characterize the clinical features in relation to age groups between pediatric and adult patient's categories.

## RESULTS

### Age-related distribution of MLs

A total of 409 cases ranged from 0 to 84 years, with a median of 33 years. There were 137 pediatric patients with the median age of 11 years (range, 0–19), and 272 adult patients with the median age of 58 years (range, 20–84).

The 137 pediatric patients included 94 cases of (< 10 years) and 43 cases within the (10–< 20 year) groups of age. The 272 adult patients included 26, 33, 39, 70, 85 and 19 cases from the groups of (20–< 30 year), (30–< 40 year), (40–< 50 year), (50–< 60 year), (60–< 70 year), and (≥ 70 year) of age, respectively.

**Figure 1:**
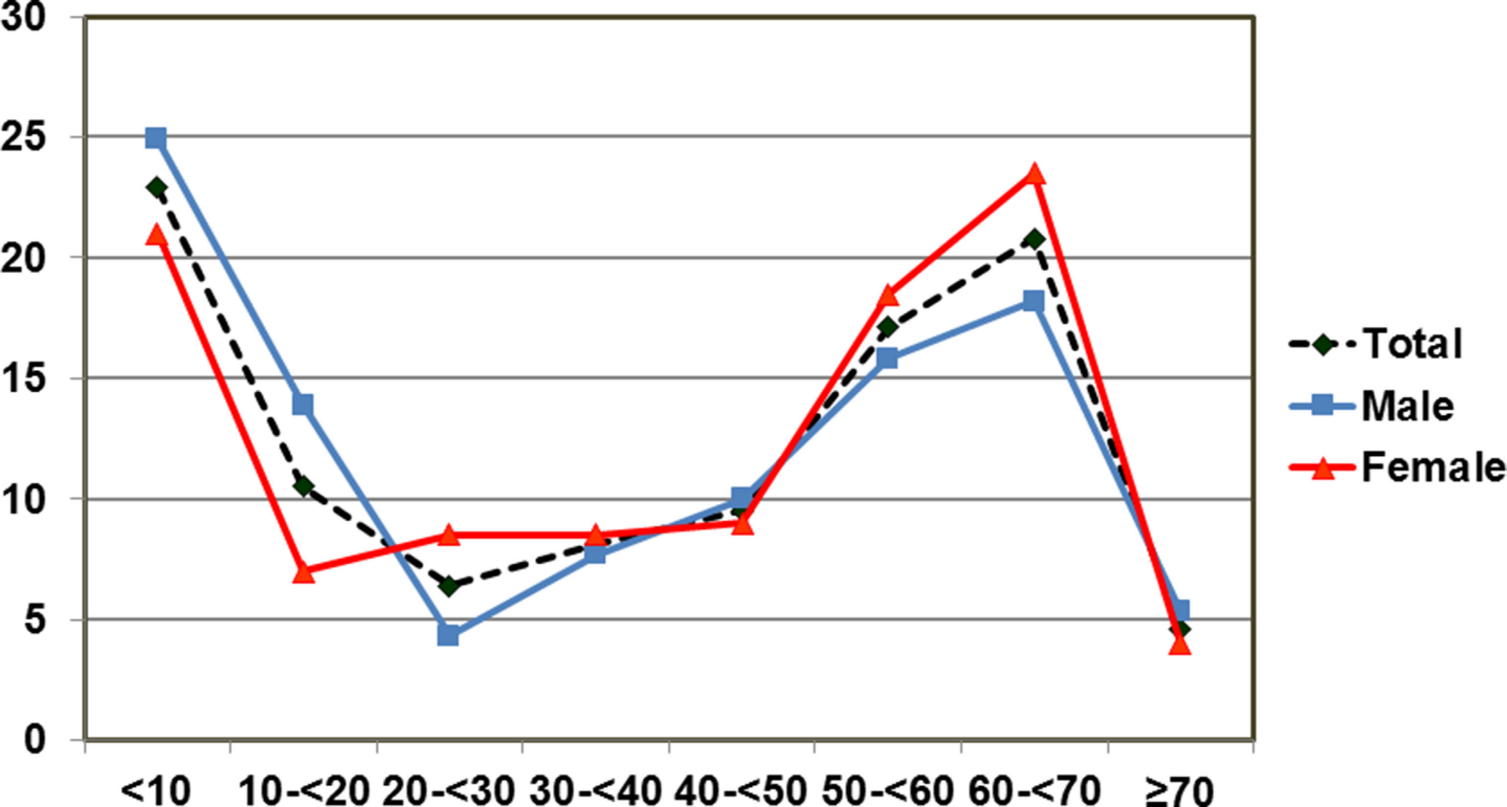
Age and sex-specific incidence of mediastinal lesions by 10-year age groups The first peak was observed in the first 10 years of life. The second peak was observed in 60-69-year age group. Both males and females showed the bimodal distribution of mediastinal lesions.

Age and gender-specific distribution of MLs were shown in Figure 1. When plotted on a frequency distribution of age at diagnosis, a bimodal distribution was shown with the peak incidences occurring within the 2 groups of age: (<10 year), and (60–< 70 year), with the incidences of 23.0% (94/409), and 20.8% (85/409), respectively.

Of the 409 cases, 51.1% (209/409) were males and 48.9% (200/409) were females. Although a similar bimodal distribution was seen in both sexes, male gender was predominant among pediatric patients, whereas female gender prevalence was shown among adults of (20–< 30 year), (50–< 60 year), and (60–< 70 year) age groups.

### Age-associated histopathological groups of MLs

**Figure 2:**
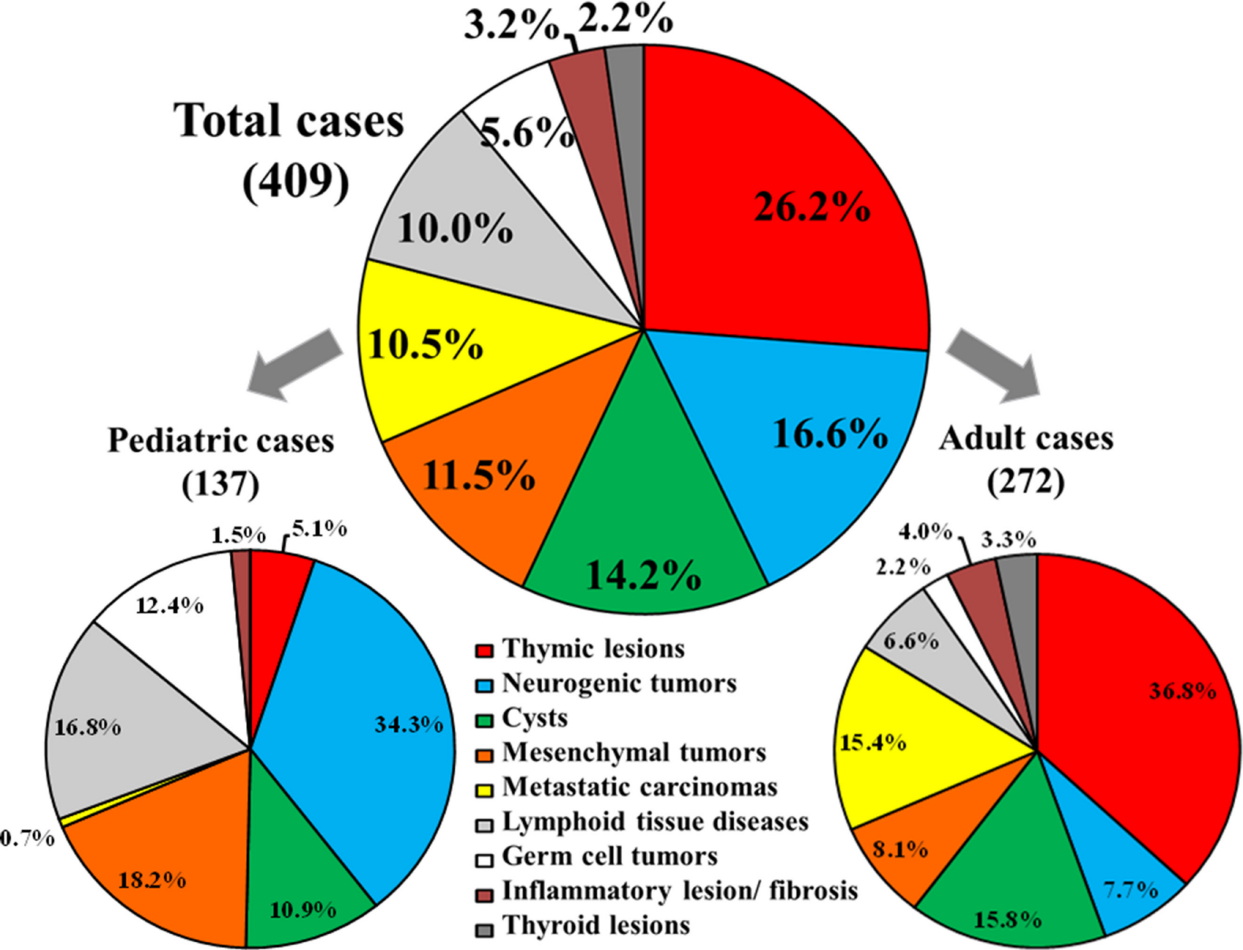
Histopathological distribution of mediastinal lesions Histopathological distribution of mediastinal lesions among the total 409 Chinese cases, and the frequency of each type of the mediastinal lesion within the 2 main age categories, including the 137 pediatric and 272 adult patients.

Age-associated histopathological profiles of MLs were shown in Figure 2. Of the total 409 cases, the most common MLs were thymic lesions (TLs), followed by NTs, cysts, mesenchymal tumors, metastatic carcinomas, lymphoid tissue diseases, germ cell tumors (GCTs), inflammatory lesion and fibrosis, and thyroid lesions, with the frequency of 26.2% (107), 16.6% (68), 14.2% (58), 11.5% (47), 10.5% (43), 10.0% (41), 5.6% (23), 3.2% (13), and 2.2% (9), respectively.

The 137 pediatric patients presented with NTs, followed by mesenchymal tumors, lymphoid tissue diseases, GCTs, cysts, TLs, inflammatory lesions and fibrosis, and metastatic carcinomas with the frequency of 34.3% (47), 18.2% (25), 16.8% (23), 12.4% (17), 10.9% (15), 5.1% (7), 1.5% (2), 0.7% (1), respectively.

Of the 272 adult patients, TLs were the most frequent diagnosis followed by cysts, metastatic carcinomas, mesenchymal tumors, NTs, lymphoid tissue diseases, inflammatory lesions and fibrosis, thyroid lesions, and GCTs with the frequency of 36.8% (100), 15.8% (43), 15.4% (42), 8.1% (22), 7.7% (21), 6.6% (18), 4.0% (11), 3.3% (9), 2.2% (6), respectively.

### Distribution of MLs-histopathological subtypes in different age groups

Based on the different age groups, NTs decreased in proportion with increasing age, while on the contrary, TLs increased in proportion. As shown in Table 1, the most common lesions in the (<10 year) age group were NTs with the frequency of 43.6% (41/94). Lymph tissue disease was the most common disease in those (10–< 20 year) and (20–< 30 year) age groups, with the frequency of 25.6% (11/43) and 26.9% (7/26), respectively. TLs occurred with their peak incidence rates in those ≥ 30 years of age. Cysts were the most common diagnosis made in the group of (50–< 60 year) of age, 25.7% (18/70), and metastatic carcinomas occurred commonly in those (60–< 70 year) and (≥ 70-year) of age groups with frequencies of 23.5% (20/85) and 26.3% (5/19), respectively.

**Table 1:** Histopathological distribution of 409 cases with mediastinal lesions in different age groups

	Pediatric patients (age in years)			Adult patients (age in years)							Total from all patients enrolled in the study
	(< 10) *n =* 94	(10–< 20) *n =* 43	Total T = 137	(20–< 30) *n =* 26	(30–< 40) n =33	(40–< 50) *n =* 39	(50–< 60) *n =* 70	(60–< 70) *n =* 85	(≥ 70) *n =* 19	Total T = 272	
Mediastinal Lesion Type	nn/n (%) nn/t (%)	nn/n (%) nn/t (%)	nn/T (%) nn/t (%)	nn/n (%) nn/t (%)	nn/n (%) nn/t (%)	nn/n (%) nn/t (%)	nn/n (%) nn/t (%)	nn/n (%) nn/t (%)	nn/n (%) nn/t (%)	nn/T (%) nn/t (%)	N *=* 409 t/N (%)
**1-Thymic lesion**	3/94 (3.2) 3/107 (2.8)	4/43 (9.3) 4/107 (3.7)	7/137 (5.1) 7/107 (6.5)	5/26 (19.2) 5/107 (4.7)	14/33 (42.4) 14/107 (13.1)	15/39 (38.5) 15/107 (14.0)	26/70 (37.1) 26/107 (24.3)	34/85 (40.0) 34/107 (31.8)	6/19 (31.6) 6/107 (5.6)	100/272 (36.8) 100/107 (93.5)	107/409 (26.2)
***A-Non-neoplastic disease***	**(3)**	**(4)**	**(7)**	**(2)**	**(8)**	**(7)**	**(11)**	**(10)**	**(1)**	**(39)**	**(46)**
Thymic hyperplasia	3	3	6	2	5	0	1	0	0	8	14
True thymic hyperplasia	0	1	1	0	1	2	0	0	1	4	5
Thymic cyst	0	0	0	0	2	5	10	10	0	27	27
***B-Neoplastic disease***	**(0)**	**(0)**	**(0)**	**(3)**	**(6)**	**(8)**	**(15)**	**(24)**	**(5)**	**(61)**	**(61)**
**Thymoma**	0	0	0	3	6	7	10	21	5	52	52
Microscopic thymoma	0	0	0	0	0	1	0	0	0	1	1
Type A	0	0	0	0	0	0	0	2	2	4	4
Type AB	0	0	0	0	0	3	1	3	0	7	7
Type B1	0	0	0	0	0	0	0	2	1	3	3
Type B2	0	0	0	3	5	1	2	3	1	15	15
Type B3	0	0	0	0	0	1	2	7	1	11	11
Type B1+B2	0	0	0	0	0	1	1	1	0	3	3
Type B2+B3	0	0	0	0	1	0	4	3	0	8	8
**Thymic carcinoma**	0	0	0	0	0	1	5	3	0	9	9
**2-Neurogenic tumor**	41/94 (43.6) 41/68 (60.3)	6/43 (14.0) 6/68 (8.8)	47/137 (34.3) 47/68 (69.1)	2/26 (7.7) 2/68 (2.9)	1/33 (3.0) 1/68 (1.5)	4/39 (10.3) 4/68 (5.9)	6/70 (8.6) 6/68 (8.8)	7/85 (8.2) 7/68 (10.3)	1/19 (5.3) 1/68 (1.5)	21/272 (7.7) 21/68 (30.9)	68/409 (16.6)
***A-Nerve sheath tumor***	**(0)**	**(1)**	**(1)**	**(2)**	**(1)**	**(3)**	**(5)**	**(6)**	**(1)**	**(18)**	**(19)**
Neurilemoma	0	1	1	2	1	3	4	5	1	16	17
Neurofibroma	0	0	0	0	0	0	1	1	0	2	2
***B-Sympathetic ganglionic tumor***	**(41)**	**(5)**	**(46)**	**(0)**	**(0)**	**(1)**	**(1)**	**(0)**	**(0)**	**(2)**	**(48)**
Ganglioneuroma	12	3	15	0	0	1	1	0	0	2	17
Ganglioblastoma	12	2	14	0	0	0	0	0	0	0	14
Neuroblastoma	17	0	17	0	0	0	0	0	0	0	17
***C-Paraganglia***	**(0)**	**(0)**	**(0)**	**(0)**	**(0)**	**(0)**	**(0)**	**(1)**	**(0)**	**(1)**	**(1)**
**3- Cyst**	13/94 (13.8) 13/58 (22.4)	2/43 (4.7) 2/58 (3.4)	15/137 (10.9) 15/58 (25.9)	4/26 (15.4) 4/58 (6.9)	5/33 (15.6) 6/58 (10.3)	3/39 (7.7) 3/58 (5.2)	18/70 (25.7) 18/58 (31)	11/85 (12.9) 11/58 (19)	2/19 (10.5) 2/58 (3.4)	43/272 (15.8) 43/58 (74.1)	58/409 (14.2)
Bronchogenic cyst	8	0	8	3	2	1	11	9	2	28	36
Mesenchymal cyst	0	1	1	1	1	0	0	2	0	4	5
Esophageal/foregut cyst	3	1	4	0	0	0	1	0	0	1	5
Parathyroid cyst	0	0	0	0	0	0	1	0	0	1	1
Others	2	0	2	0	2	2	5	0	0	9	11
**4-Mesenchymal tumor**	18/94 (19.1) 18/47 (38.3)	7/43 (16.3) 7/47 (14.9)	25/137 (18.2) 25/47 (53.2)	1/26 (3.8) 1/47 (2.1)	6/33 (18.2) 6/47 (12.8)	5/39 (12.8) 5/47 (10.6)	4/70 (5.7) 4/47 (8.5)	4/85 (4.7) 4/47 (8.5)	2/19 (10.5) 2/47 (4.3)	22/272 (8.1) 22/47 (46.8)	47/409 (11.5)
***A-Benign tumor***	**(17)**	**(4)**	**(21)**	**(1)**	**(4)**	**(5)**	**(1)**	**(4)**	**(1)**	**(16)**	**(37)**
Lipoma	2	0	2	0	1	0	1	0	0	2	4
Lymphangioma	13	1	14	0	1	1	0	1	0	3	17
Hemangioma	1	2	3	0	1	1	0	1	1	4	7
Leiomyoma	0	0	0	0	0	0	0	1	0	1	1
Mesothelioma	0	0	0	0	0	0	0	1	0	1	1
Solitary fibrous tumor	0	0	0	1	0	1	0	0	0	2	2
Fibromatosis	1	1	2	0	1	2	0	0	0	3	5
***B-Malignant tumor***	**(1)**	**(3)**	**(4)**	**(0)**	**(2)**	**(0)**	**(3)**	**(0)**	**(1)**	**(6)**	**(10)**
Primitive neuroectodermal tumor	0	3	3	0	0	0	0	0	0	0	3
Liposarcoma	0	0	0	0	0	0	0	0	1	1	1
Fibrousarcoma	1	0	1	0	0	0	1	0	0	1	2
Chondrosarcoma	0	0	0	0	0	0	1	0	0	1	1
Low grade fibromyxoid sarcoma	0	0	0	0	1	0	0	0	0	1	1
Undifferentiated pleomorphic sarcoma	0	0	0	0	0	0	1	0	0	1	1
Rhabdomyosarcoma	0	0	0	0	1	0	0	0	0	1	1
**5-Metastatic carcinoma**	0	1/43 (2.3) 1/43 (2.3)	1/137 (0.7) 1/43 (2.3)	1/26 (3.8) 1/43 (2.3)	1/33 (3.0) 1/43 (2.3)	6/39 (15.4) 6/43 (14.0)	9/70 (12.9) 9/43 (20.9)	20/85 (23.5) 20/43 (46.5)	5/19 (26.3) 5/43 (11.7)	42/272 (15.4) 42/43 (97.7)	43/409 (10.5)
Squamous cell carcinoma	0	0	0	0	0	2	4	5	1	12	12
Adenocarcinoma	0	0	0	0	1	1	2	3	0	7	7
Neuroendocrine carcinoma	0	0	0	0	0	0	1	4	1	6	6
Others	0	1	1	1	0	3	2	8	3	17	18
**6-Lymphoid tissue disease**	12/94 (12.8) 12/41 (29.3)	11/43 (25.6) 11/41 (26.8)	23/137 (16.8) 23/41 (56.1)	7/26 (26.9) 7/41 (17.2)	6/33 (18.2) 6/41 (14.6)	3/39 (7.6) 3/41 (7.3)	1/70 (1.4) 1/41 (2.4)	1/85 (1.2) 1/41 (2.4)	0	18/272 (6.6) 18/41 (43.9)	41/409 (10.0)
***A-Lymphoma***	**(12)**	**(11)**	**(23)**	**(7)**	**(5)**	**(2)**	**(1)**	**(1)**	**(0)**	**(16)**	**(39)**
T- lymphoblastic lymphoma	10	9	19	2	2	1	0	0	0	5	24
Classical Hodgkin’s Disease	2	2	4	1	1	0	0	0	0	2	6
Primary Mediastinal LBCL	0	0	0	4	1	1	0	0	0	6	6
DLBCL	0	0	0	0	1	0	0	1	0	2	2
MALT lymphoma	0	0	0	0	0	0	1	0	0	1	1
***B-Castleman disease***	**(0)**	**(0)**	**(0)**	**(0)**	**(1)**	**(1)**	**(0)**	**(0)**	**(0)**	**(2)**	**(2)**
**7- Germ cell tumor**	7/94 (7.4) 7/23 (30.5)	10/43 (23.3) 10/23 (43.5)	17/137 (12.4) 17/23 (73.9)	5/26 (19.2) 5/23 (21.7)	0	1/39 (2.6) 1/23 (4.3)	0	0	0	6/272 (2.2) 6/23 (26.1)	23/409 (5.6)
***A-Teratoma***	**(7)**	**(8)**	**(15)**	**(4)**	**(0)**	**(1)**	**(0)**	**(0)**	**(0)**	**(5)**	**(20)**
Mature teratoma	6	7	13	2	0	0	0	0	0	2	15
Immature teratoma	1	1	2	0	0	0	0	0	0	0	2
Teratocarcinoma	0	0	0	2	0	1	0	0	0	3	3
***B-Seminoma***	**(0)**	**(0)**	**(0)**	**(1)**	**(0)**	**(0)**	**(0)**	**(0)**	**(0)**	**(1)**	**(1)**
***C-Mixed germ cell tumor***	**(0)**	**(2)**	**(2)**	**(0)**	**(0)**	**(0)**	**(0)**	**(0)**	**(0)**	**(0)**	**(2)**
**8-Inflammatory and fibrosis**	0 0	2/43 (4.7) 2/13 (15.4)	2/137 (1.5) 2/13 (15.4)	1/26 (3.8) 1/13 (7.7)	0	1/39 (2.6) 1/13 (7.7)	4/70 (5.7) 4/13 (30.8)	4/85 (4.7) 4/13 (30.8)	1/19 (5.3) 1/13 (7.7)	11/272 (4.0) 11/13 (84.6)	13/409 (3.2)
**9- Thyroid lesion**	0	0	0	0	0	1/39 (2.6) 1/9 (11.2)	2/70 (2.9) 2/9 (22.2)	4/85 (4.7) 4/9 (44.4)	2/19 (10.5) 2/9 (22.2)	9/272 (3.3) 9/9 (100)	9/409 (2.2)

DLBCL, diffuse large B-cell lymphoma; MALT, mucosa associated lymphoid tissue; n, number of cases per a group of age; nn, number of cases with the same mediastinal lesion within a group of age; N, number of all cases enrolled in the study; t, total number of cases of a particular mediastinal lesion in the study; T, sum of cases of different age groups in pediatric or adult category.

The 107 cases with TLs included 46 cases of non-neoplastic diseases and 61 cases of neoplastic diseases. Of the 107 cases of TLs, thymic hyperplasia and true thymic hyperplasia cases were seen in both pediatric and adult patients, whereas thymic cyst, thymoma and thymic carcinoma occurred predominantly among adult patients of (40–70 year) of age.

The 68 cases of NTs were classified into nerve sheath tumors, 27.9% (19/68), sympathetic ganglionic tumors, 70.6% (48/68) and paraganglia, 1.5% (1/68). Neuroblastoma occurred exclusively in those younger than 5 years, ganglioblastoma and ganglioneuromas occurred mainly in (< 10 year) age group, whereas, neurilemmoma (schwannoma) and neurofibroma occurred frequently in adult patients.

Among the 58 cases with the diagnosis of cysts, bronchogenic cyst was the most common type with a total frequency of 62.1% (36/58), and was predominant among adult patients of (50–< 60 year) and (60–< 70 year) age groups, with frequencies of 30.6% (11/36), and 25% (9/36), respectively. However, in children of (< 10 year), it was found in 22.2% (8/36).

Mesenchymal tumors commonly presented with benign tumors, 78.7% (37/47), followed by malignant tumors, 21.3% (10/47). Of the 37 cases of benign tumors, lipoma and lymphangioma were predominantly seen in pediatric patients, while hemangioma, leiomyoma, mesothelioma, solitary fibrous tumors and fibromatosis were found commonly in adult patients. Of the 10 cases of malignant tumors, 3 cases of primary neuroectodermal tumors, were reported in pediatric patients of (10–< 20 year) group of age, whereas other types of sarcomas were predominantly diagnosed in adult patients.

Lymphoma accounted for 95.1% (39/41) of the lymphoid tissue diseases. T-cell lymphoblastic lymphoma represented 61.5% (24/39) and classical Hodgkin's disease (HD) represented 15.4% (6/39), and were predominantly diagnosed in pediatric patients, whereas the primary mediastinal large B-cell lymphoma was the main subtype in adult patients of (20–< 50 year) groups of age, and represented 15.4% (6/39). Two cases of Castleman disease were documented in adult patients of the (30–< 50 year) of age.

GCTs included teratoma in 87% (20/23), seminoma in 4.3% (1/23) and mixed GCTs in 8.7% (2/23). Mature teratoma, immature teratoma and mixed GCTs occurred commonly in pediatric patients, in contrast, teratocarcinoma and seminoma were mainly seen in male adult patients of (20–< 30 year) group of age.

Inflammatory lesions and fibrosis were found in pediatric and adult patients, while thyroid lesions and metastatic carcinoma were rarely seen in pediatric category.

### Clinical features and MLs in different age groups

As shown in Table 2, most of the cases 41.6%, (170/409) were asymptomatic at the time of diagnosis, including 50 pediatric and 120 adult patients. The most common symptoms were cough, 23.9% (98/409), followed by chest pain, 15.6% (64/409) and fever, 8.3% (34/409). Cases that presented with myasthenia gravis-associated thymoma were found in 3.2% (13/409). Among the 137 cases of pediatric patients, cough, fever, and dyspnea were the most common symptoms. Whereas among the 272 cases of adult patients, cough, chest pain and chest oppression were the main symptoms.

**Table 2:** Clinical features and lesion locations of 409 cases with mediastinal lesions in different age groups

	Pediatric patients (age in years)			Adult patients (age in years)							*P*-value (Pediatric vs. adults)	All enrolled patients N = 409 (%)
	(< 10) *n* = 94	(10–< 20) *n* = 43	Total T = 137 (%)	(20–< 30) *n* = 26	(30–< 40) *n* = 33	(40–< 50) *n* = 39	(50–< 60) *n* = 70	(60–< 70) *n* = 85	(≥ 70) *n* = 19	Total T = 272 (%)		
***Gender, Male: Female***	52:42	29:14	81:56	9:17	16:17	21:18	33:37	38:47	11:8	128:144	0.02	209:200
***Asymptomatic***	38	12	50/137 (36.5)	11	17	14	36	32	10	120/272 (44.1)	0.14	170/409 (41.6)
***Symptomatic***												
Cough	33	14	47/137 (34.3)	4	6	5	11	22	3	51/272 (18.7)	< 0.01	98/409 (23.9)
Chest pain	6	7	13/137 (9.5)	8	4	10	13	14	2	51/272 (18.7)	0.02	64/409 (15.6)
Fever	18	5	23/137 (16.8)	1	3	0	3	3	1	11/272 (4.0)	< 0.01	34/409 (8.3)
Chest oppression	0	1	1/137 (0.7)	3	4	7	3	8	4	29/272 (10.6)	< 0.01	30/409 (7.3)
Dyspnea	11	4	15/137 (10.9)	1	1	1	3	7	0	13/272 (4.8)	0.02	28/409 (6.8)
Myasthenia graves	0	0	0	1	0	3	3	5	1	13/272 (4.8)		13/409 (3.2)
***Location in mediastinum***												
Superior	20	5	25/137 (18.2)	3	4	6	9	9	6	37/272 (13.6)	0.22	62/409 (15.2)
Anterior	26	28	54/137 (39.4)	20	24	25	40	51	10	170/272 (62.5)	< 0.01	224/409 (54.7)
Middle	4	5	9/137 (6.6)	1	3	4	9	15	2	34/272 (12.5)	0.06	43/409 (10.5)
Posterior	44	5	49/137 (35.8)	2	2	4	12	10	1	31/272 (11.4)	< 0.01	80/409 (19.6)

n, number of cases per a group of age; N, number of all cases enrolled in the study; t, total number of cases of a particular mediastinal lesion in the study; T, sum of cases of different age groups in pediatric or adult category.

Concerned with the location of the MLs, from the total 409 cases, the most common location was the anterior mediastinum; 54.7% (224/409), followed by posterior; 19.6% (80/409), superior; 15.2% (62/409), and the middle mediastinum; 10.5% (43/409).

## DISCUSSION

In this report, we retrospectively reviewed 409 patients with MLs in a single Chinese institution. Adult cases were around double the pediatric cases, and as reported previously, the pediatric series showed male gender predominance [9]. This considerably high incidence of males is likely related to the high proportion of NTs, lymph tissue diseases, mesenchymal tumors and GCTs, which preferentially occur in males [10, 11].

TLs, NTs, and cysts made up 57% of our encountered MLs among the total 409 cases. A significantly higher frequency was found for NTs, GCTs, mesenchymal tumors, and lymphatic lesions, (*p* < 0.01) for all, in our pediatric population compared to adults. On the contrary, TLs and metastatic carcinomas were significantly higher in adults compared to pediatric age group, (*p* < 0.01) for both. Within the pediatric age group of (< 10 year), a higher proportion of MLs were found due to the increased cases of NTs (43.6%), and the pediatric NTs represented 69.1% of all the 68 cases of NTs from the total 409 patients. Whereas in adult cases, the peak incidence was found in the group of (60–< 70 year), which was made by the increased number of TLs and metastatic carcinomas.

TLs were the most common MLs among the total of 409 patients, with a frequency of 26.2%, comparable to the frequencies of USA; 28.4% (*p* = 0.56) [5] and 31% (*p* = 0.33) [12], and that of Japan 31.5% (*p* = 0.09) [6]. The highest frequency of TLs was among adult category, similar to Japan [6] and USA [12]. In our adult's subset, TLs were diagnosed between 4th–7th decades of life, and thymoma was the main type of TLs in 52% of adult cases [13]. In contrast, TLs in our pediatric category, made up only 5.1% of MLs (all cases were found to be thymic hyperplasia), comparable to that of Japan 4% (*p* = 0.39) and USA 10% (*p* = 0.72) [6, 12]. Type B thymomas were often associated with myasthenia gravis than other type A and AB in our cases [13, 14].

NTs were the second MLs type encountered in the total series of our cases, 16.6%, similar to that reported in Japan 16.9% [6]. Pediatric category showed that NTs were the commonest MLs, comprised of 34.3%, in agreement with Azarow et al. with the frequency of 34% [5], as well as Simpson et al. [15] and Whooley et al. [12] of 24% (*p* = 0.07) and 50% (*p* = 0.17), respectively. Our figures of NTs in terms of order and frequency from total Chinese series were similar to that of total Japanese cases, and NTs were the commonest MLs in the pediatric subsets in both studies. However, our frequency of 34.3% among pediatric subset, was lower than that of Japan 46%, to reach a significant level (*p* = 0.04). Taken together, the higher frequency of ganglioneuroma in the Japanese pediatric group (more than double of our cases), and the definition of Japanese pediatric group below 15 years of age, could explain the significant difference. The two major categories of sympathetic nervous system and peripheral nerve sheath had a definite relationship with age. In our pediatric patients, the vast majority presented with the sympathetic ganglionic tumors in children (< 10 years), including neuroblastomas, ganglioneuromas, or ganglioblastoma, while infants mostly had neuroblastomas [7]. On the other hand, among adult category, NTs were the fifth most common MLs, similar in order of frequency to that of Japanese adults, with comparable figures, 7.7% to 11% (*p* = 0.11) [6] and to Azarow et al., of 12.3% (*p* = 0.10) [5]. In contrast to pediatric series, the frequency of nerve sheath tumors increased in adult patients, as reported previously [16]. Thus, age seems to be the most important clinical parameter for distinguishing between histopathological types and rate of malignancy in NTs [17].

The frequency of cystic MLs 14.2% in the total of our series was comparable to that reported in the literature 15–20% [1]. The prevalence of cysts in the mediastinum in adults was higher than that in children. We confirmed the previously reported higher frequencies of bronchogenic cysts than other types in our series, among mediastinal cysts, 62.1% (36/58), with a higher frequency in adults 77.8% (28/36) compared to pediatric counterpart 22.2% (8/36) [18].

Mesenchymal tumors occurred throughout childhood as well as adulthood. Lymphangioma was the type of benign tumors seen in children and predominantly in those younger than 3 years of age, 76.5% (13/17) [19]. The distribution pattern of some soft tissue sarcomas differed significantly between adults and children, primary neuroectodermal tumor was most common in children whilst other types of sarcoma were seen typically in adults [20, 21].

Lymphoma is common among pediatric patients. In our study, T-lymphoblastic lymphoma predominantly occurred in children younger than 10 years, whereas primary mediastinal large B-cell lymphoma occurred in adults between the 3^rd^–5^th^ decades of age, whilst classical HD affected both children and adults [22–25]. The frequency of lymphomas among our total series 10%, was comparable to that of Japan 12.3% (*p* = 0.24), but significantly lower than that of Davis et al., 16% (*p* = 0.01) [7], and of Whooley et al., 19%, (*p* = 0.01) [12]. Concerned with our pediatric category, lymphoma was within the range of previous reports [26], Japan 13% (*p* = 0.39) [6], USA 17.3% (*p* = 0.16) [12], and Turkey 18.3% (*p* = 0.73) [27], however, it was significantly lower than USA reports; 32.3% (*p* = 0.01) [3] and 55% (*p* < 0.01) [9], as well as that of Simpson et al., 49.6% (*p* < 0.01) [15]. Likewise, the prevalence of lymphomas in our adult subset 7%, was significantly lower than that of USA reports 21% and 55%, both (*p* < 0.01) [5, 9]. Of note, some of these reports were dating back in 1970s, or even older, where the diagnostic facilities were limited. Furthermore, our results represented one institution in China.

The prevalence of GCTs in our total series was 5.6%, significantly lower than that of previous reports of Japan 16.1% (*p* < 0.01) [6], Davis et al., 11% (*p* < 0.01) [7], and of USA 23% (*p* < 0.01) [12]. As reported previously, mediastinal GCTs represented more commonly in children than adults. Our study showed that most mature teratomas were seen in children, whereas seminoma and teratocarcinoma occurred in adults [28, 29]. The frequency of GCTs in pediatric series, 12.4% was within the range of previous studies in USA; 5% (*p* = 0.55) [12], 8.1% (*p* = 0.51) [3], and 18% (*p* = 0.69) [9], and in Japan 19% (*p* = 0.17) [6]. However, the prevalence of GCTs in our adult series 2.2%, was significantly lower than Japan 16% (*p* < 0.01) [6], and lower than those reports from USA 15% and 12.3%, both (*p* < 0.01) [5, 9].

Benign thyroid nodules were the most common changes in the mediastinal thyroid gland, and occurred commonly in female adults. Metastatic carcinomas of the lung and that of the breast to the mediastinal lymph nodes were centered in the middle compartment of the mediastinum. In our study, thyroid, parathyroid lesions and metastatic carcinomas occurred in adult patients.

In combined adult and pediatric population, we found the frequencies for anterior, middle, and posterior MLs as follows: 54.7%, 10.5%, and 19.6%, respectively, comparable to those in literature [7]. In the adult subset, anterior MLs comprised 62.5%, and in pediatric cases, posterior MLs made up 35.8%, such difference was comparable to previous reports [3, 5, 6, 30]. Our results agreed with the previous reports that MLs in pediatric patients presented with a significantly higher frequency in the posterior mediastinum (35.8%, 49/137 vs. 11.4%, 31/272) (*p* < 0.01), and a significantly lower frequency in the anterior mediastinum (39.4%, 54/137 vs. 62.5%, 170/272) (*p* < 0.01), and a trend toward a lower frequency in the middle mediastinum (*p* = 0.06) compared to adult patients [31].

Among pediatric category, the higher frequency of malignancy 52.1% was reported in the first decade of life, however, among the whole age spectrum, the highest rate of malignant cases was reported in the 8th decade of life 57.9%. The mean frequency of the malignant cases was almost similar in both pediatric and adult categories (48.2% in pediatric cases) and (48.5% in adult cases), comparable to previous reports [5].

Overall, 41.6% (170/409) were asymptomatic, however, pediatric patients showed a significantly higher incidence of cough, dyspnea, fever and a lower incidence of chest pain or oppression than adult counterpart, as shown in Table 2, similar to previous reports [5–7, 32]. It might be that symptoms due to compression or direct invasion into an adjacent structure caused the high incidence of respiratory symptoms in pediatric population compared to the adult population. The underlying tumor subtypes might explain the differences; NTs, lymph tissue disease and GCTs peaked in incidence in pediatric group, which commonly presented with respiratory symptoms. Generally, large MLs, including malignant GCTs or lymphomas, tended to cause respiratory symptoms of dyspnea and chest pain regardless of the malignancy.

The limitation of this study is the number of cases from a single institution, which is relatively small to allow the detailed analysis. More detailed studies are needed to understand the MLs distribution in different age categories and its relation with clinicopathological features.

## MATERIALS AND METHODS

Cases were obtained from the files of a single institution (Chinese institution of Xinhua Hospital). A total of 409 cases with MLs were retrospectively reviewed between August 2012 and July 2016. Clinical data concerning age at presentation, gender, symptoms, treatment were retrieved from the medical records. We arbitrarily defined pediatric patients (children and adolescents) as < 20 years of age, and adults as ≥ 20 years of age. The patients were categorized into eight different age groups: (< 10 years), (10–< 20 years), (20–< 30 year), (30–< 40) year, (40–< 50) year, (50–< 60 year), (60–< 70 year), and (≥70 year) of age. Written informed consent was previously obtained from each patient and/or guardians according to the guidelines of the Declaration of Helsinki.

MLs were identified by imaging studies such as chest X-ray, computed tomography (CT), magnetic resonance imaging (MRI), and fluorodeoxyglucose (FDG) positron emission tomography (PET)-scan, and imaging findings as well as medical and surgical records were reviewed. The mediastinum was divided into superior and inferior compartments by an imaginary line traversing the manubriosternal joint and the lower surface of the fourth thoracic vertebra on the lateral X-ray image. The inferior compartment was further subdivided into three compartments: the middle mediastinum, which contains the pericardium and its contents as well as the major vessels and airways; the anterior mediastinum, which lies anterior to the middle mediastinum and posterior to the sternum; and the posterior mediastinum, which lies posterior to the middle mediastinum and anterior to the thoracic vertebral column [33].

Hematoxylin and eosin-stained sections from routinely fixed, paraffin-embedded tissues were examined. Histopathological diagnosis was established according to the morphological and immunohistochemical evaluations. Data obtained were classified into different groups on the bases of histopathological classification of the MLs [34].

Contingency tables were analyzed using Pearson Chi-square statistic. All tests were two-sided with *p*-value < 0.05, were regarded as statistically significant. The software of SPSS version 17.0 (SPSS Inc., Chicago, IL, USA) was used for statistical calculations.

## CONCLUSIONS

Our study demonstrated that age spectrum influenced the histopathological distribution and the clinical presentation in MLs in Chinese series of patients. Such differences should be considered in the differential diagnosis and therapeutic approach for adult and pediatric patients with MLs. Furthermore, our study was comparable to the literature in terms of MLs frequencies.
